# Sleep Attributes Influencing Cardiovascular Morbidity Among Young Adults Pursuing Professional Courses in Dehradun, India: A Cross-Sectional Study

**DOI:** 10.7759/cureus.52647

**Published:** 2024-01-21

**Authors:** Hitesh Nautiyal, Debabrata Roy, Anupama Arya, Sonam Maheshwari, Pratik Agarwal, Neil Patel, Yashendra Sethi

**Affiliations:** 1 Community Medicine, Government Doon Medical College, Dehradun, IND; 2 Research, PearResearch, Dehradun, IND; 3 Medicine, Lokmanya Tilak Municipal Medical College, Mumbai, IND; 4 Medicine and Surgery, Gujarat Medical Education & Research Society (GMERS) Medical College, Himmatnagar, IND; 5 Internal Medicine, Government Doon Medical College, Dehradun, IND

**Keywords:** sleep problems, sleep duration, sleep patterns, young adults, cardiovascular disease

## Abstract

Cardiovascular diseases (CVDs) are evolving as a cause of mortality and morbidity among young adults. Young adults, particularly those pursuing professional courses in colleges, face unique challenges that may influence their risk of developing CVD. Despite screening guidelines, CVD risk factors often go undetected in the young population, highlighting the need for increased awareness among adolescents. Sleep is an essential indicator of well-being, and its impact on cardiovascular risk factors is increasingly being recognized. An observational cross-sectional study was conducted among young adults aged 18 to 24 years pursuing professional courses in Dehradun, Uttarakhand, India. A total of 156 participants were recruited through multistage, systematic random sampling, and snowball sampling. Data on sleep patterns and cardiovascular morbidity were collected using a pretested questionnaire. Among the study participants, 46.8% reported having less than four hours of sleep on average, and 25% were suffering from sleep problems. The prevalence of diagnosed CVDs was low, with 6.14% reporting arrhythmias and 3.84% reporting hypertension. An association was found between sleep duration, sleep problems, and the presence of CVDs. Participants with shorter sleep durations and more severe sleep problems had a higher prevalence of CVDs. The findings suggest that sleep duration and sleep problems may be modifiable risk factors for CVDs among young adults. Effective health promotion activities focusing on behavior and lifestyle modifications are essential to preventing CVDs from an early age. The study emphasizes the importance of early intervention and health promotion strategies to reduce CVD risk factors in this population. Community-based research and behavior change communication initiatives are recommended to promote healthy sleep habits and prevent cardiovascular diseases among young adults beyond the college setting.

## Introduction

Non-communicable diseases (NCDs) are responsible for over 60% of all deaths. Common NCDs include cardiovascular disease (CVD), various malignancies, chronic respiratory conditions, and diabetes [1]. In low- and middle-income nations, mortality from CVD, diabetes, and chronic obstructive pulmonary disease accounts for more than 80% of all deaths [2]. Young adults are unique because they have outgrown their adolescent dependence but have not yet assumed all the duties of adulthood. During this transitional phase, they may be more susceptible to adopting risky behaviors due to reduced parental supervision, increasing independence, and financial instability [3]. Specifically, young adults pursuing professional courses in colleges are more prone to adopt habits that increase the risk of CVD [4]. Despite screening guidelines for all individuals over the age of 20, these conditions often go undetected in the young population [5-7]. Further, most young people are unaware of the risk factors [8]. Therefore, it is crucial to raise awareness among adolescents about the risks of CVD to eradicate the disease among young individuals [9]. The current situation in India shows that the youth of this nation are at risk for CVD-related diseases. Long-term CVD risk can be predicted by the existing risk factors in young people. Despite CVD risk factors being persistently high among youth, they are underappreciated and will continue to rise as people get older [10].

Sleep is an essential indicator of well-being and health in humans. Recent studies suggest that chronic insomnia or an altered sleep cycle can exacerbate some cardiovascular risk factors, such as the development of diabetes, high blood pressure, weight gain, obesity, and an elevated resting heart rate [11]. It has been proposed that maintaining a healthy sleep pattern is associated with reduced risks of CVDs [12]. Given the ongoing epidemiological transition to non-communicable and lifestyle diseases, including CVDs, it becomes pertinent to study the burden, if any, among young adults.

Young adults pursuing professional courses in colleges constitute an important and understudied population in India, as they are exposed to distinct lifestyle situations, behaviors, and factors that may influence their risk of developing CVD. In the state of Uttarakhand, there is a lack of published scientific research focusing on young adults concerning their socio-demographic, behavioral, and epidemiological perspectives. Therefore, conducting research in this area is crucial, and this study aims to assess sleep patterns and explore the situations, behaviors, and factors that contribute to the risk of CVD morbidity among young adults in selected professional colleges in the district of Dehradun. The study aims to assess sleep attributes influencing cardiovascular morbidity among young adults pursuing professional courses in the district of Dehradun, Uttarakhand, India. 

## Materials and methods

Study type and population

This is an observational, descriptive, and cross-sectional study, approved by the Institutional Ethics Committee of Government Doon Medical College and Hospital, Dehradun, Uttarakhand, India (approval no. GDMC/IEC/2022/05). It was conducted by collecting data over 12 months between 2022 and 2023 among sampled young adults, aged between 18 and 24, pursuing professional courses in the Dehradun district. Participants' informed consent was received under the supervision and guidance of resources from the Department of Community Medicine, Government Doon Medical College. All personal information of the study participants was kept confidential.

Study sampling

A multistage, systematic random sampling method was employed. Stage one involved identifying colleges and institutes offering varied professional courses (health sciences, engineering, law, pharmacy, etc.) in the district. They were stratified as separate sampling frames based on the professional course offered. Sampling units from the respective strata or sampling frame were chosen by systematic random sampling with a sampling interval (SI) of 4, i.e., every fourth college or institute was chosen from the respective frame. The sampled colleges and institutes thus calculated were 12.

Stage two entailed the calculation of the sample size. Considering cardiovascular morbidity prevalence from across different Indian study settings, including Uttarakhand [13-16] to be an estimated average of 10%, the suggested sample size as calculated was 152, considering 10% non-respondents, an allowable absolute error or precision of 5%, and a 95% confidence interval.

Stage three was when study subjects from each sampled college or institute were chosen through a snowball sampling technique in which the researcher selected a volunteer who, in his turn, had randomly selected other volunteers. Out of a suggested sample of 152, study subjects chosen from each of the sampled 12 colleges or institutes were 13, i.e., 13 study subjects from each sampled college or institute were chosen to participate in the study. Thus, the final sample size is 13 x 12 = 156.

Inclusion/exclusion criteria, study tools, and statistical analysis

We included young adults between 18 and 24 years of age who were willing to participate and present at the time of data collection. The respondents who were unwilling to participate and those who were not present at the time of data collection were excluded. A pretested, predesigned instrument was administered to the study subjects to elicit data. All collected data was compiled, tabulated, and analyzed using SPSS Statistics version 22.0 (IBM Corp., Armonk, NY, USA) and Microsoft Excel 2007 (Microsoft Corp., Redmond, WA, USA). The percentage was calculated for all the variables; the Chi-square test was employed to evaluate whether a finding or association was statistically significant or not. The significance level was assumed to be p <0.05.

## Results

A majority (58.9%) of the participants belonged to the age range of 21 to 24, with 58.3% of participants being male and 41.7% female. Around 99.4% of participants were unmarried. A majority of the participants belonged to the Hindu religion (91.7%), followed by Muslims at 4.5% and Sikhs at 2.6%. About 69.2% of young adults belonged to a nuclear family, with 42.9% of them belonging to the upper social class, followed by 24.4% in the upper-middle and 23.1% in the middle, per the modified BG Prasad classification (Table 1).

**Table 1:** Demographic characteristics of study participants

Demographic Variable	Frequency (n=156)	Percentage
Age		
18-20	64	41.0
21-24	92	58.9
Gender		
Male	91	58.3
Female	65	41.7
Marital status		
Unmarried	155	99.4
Married	1	0.6
Religion		
Hindu	143	91.7
Muslim	7	4.5
Sikh	4	2.6
Others	2	1.3
Family type		
Nuclear	108	69.2
Joint	40	25.6
Three-generation family	8	5.1
Total family members		
<4	68	43.6
5-10	74	47.4
11-15	8	5.1
>16	6	3.8
Social class		
Upper class	67	42.9
Upper-middle	38	24.4
Middle	36	23.1
Lower-middle	14	9.0
Lower class	1	0.6

Table 2 represents self-reported diseases involving the cardiovascular system by the study participants, where a maximum of 10 (6.14%) participants had arrhythmias, six (3.84%) had hypertension, four (2.56%) had rheumatic heart disease, two (1.28%) had valvular heart disease, and four (2.56%) had other problems involving the cardiovascular system.

**Table 2:** Distribution of study subjects by diagnosed CVDs (n=156) CVDs: Cardiovascular diseases

Diseases	Frequency: n (%)	Duration since diagnosis (mean±SD)
Coronary artery disease (myocardial infarction)	0	0
Cerebrovascular disease (stroke)	0	0
Peripheral heart disease	0	0
Rheumatic heart disease	4 (2.56%)	2.8±0.63
Valvular heart disease	2 (1.28%)	1.3±0.12
Arrhythmias (abnormal heart rhythms)	10 (6.14%)	3.1±0.71
Angina	0	0
Heart failure	0	0
Deep vein thrombosis/pulmonary embolism	0	0
Cardiomyopathies	0	0
Hypertension	6 (3.84%)	2.9±0.88
Others	4 (2.56%)	1.9±0.28

It is evident from Table 3 that a majority, i.e., 46.8% of study participants, had less than four hours of sleep and took less than 30 minutes to fall asleep. Only 9% had a sleep of seven to eight hours. None of the subjects slept for more than eight hours on average in 24 hours. Most of the participants (47.4%) ‘tried to fall asleep’; 25% of participants ‘thought of college matters’ while awake in bed; 49.4% ‘woke up in the morning feeling quite tired’; and 30.1% ‘woke up in the night complaining of nightmares or frightening dreams and feeling quite anxious’. Around 17.3% had ‘difficulty falling asleep or interrupted sleep’ followed by 13.5% experiencing 'snoring', 5.8% having ‘insomnia’, and 3.2% reporting ‘obstructive sleep apnea’. Further, 25% of the respondents were 'suffering from sleep problems, whereas as much as 60.9% ‘did not know’ about the severity of their sleep problems. Table 4 shows that differences in the proportions of study participants concerning sleep duration (hours of sleep on average in 24 hours) and sleep problem(s) were significant, implying a statistically significant association between sleep duration and sleep problems among study participants and the prevalence of CVD among them. 

**Table 3:** Distribution of study subjects by the factors, behaviors, and situations influencing sleep (n=156)

Factors/behaviors/situations	Frequency: n (%)
How many hours of sleep do you have on average in 24 hours?	
0-4 hours	73 (46.8%)
5-6 hours	69 (44.2%)
7-8 hours	14 (9%)
More than 8 hours	0
On average how long does it take you to fall asleep?	
Less than 15 minutes	52 (33.3%)
Less than 30 minutes	73 (46.8%)
Less than 60 minutes	19 (12.2%)
More than 1 hour	12 (7.7%)
When you are in bed awake, what do you think about?	
Trying to fall asleep	74 (47.4%)
Family matters	23 (14.7%)
College matters	39 (25%)
Others	20 (12.8%)
How often do you have trouble getting off to sleep?	
Never	42 (26.9%)
Less than once a month	28 (17.9%)
About once a month	24 (15.4%)
Two to four times a month	39 (25%)
Many times a week	15 (9.6%)
Daily	8 (5.1%)
Do you wake up in the morning well-rested?	
Yes	105 (67.3%)
No	51 (32.7%)
Do you wake up in the morning feeling quite tired?	
Yes	77 (49.4%)
No	79 (50.6%)
Do you wake up at night complaining of nightmares or frightening dreams and feel quite anxious?	
Yes	47 (30.1%)
No	109 (69.9%)
Do you wake up at night screaming in terror?	
Yes	26 (16.7%)
No	130 (83.3%)
Do you experience any of the following?	
Snoring	21 (13.5%)
Apneic episodes	5 (3.2%)
Insomnia	9 (5.8%)
Difficulty falling asleep or interrupted sleep	27 (17.3%)
Don't know	93 (59.6%)
Are you suffering from sleep problems?	
Yes	39 (25%)
No	117 (75%)
How severe do you consider your sleep problems to be?	
Mild	30 (19.2%)
Moderate	16 (10.3%)
Severe	2 (1.3%)
Don't know	95 (60.9%)
Absent	13 (8.3%)

**Table 4:** Association between the key factors, behaviors, and situations influencing sleep and CVD (n=156) CVD: Cardiovascular disease

Sleep duration/sleep problems		Cardiovascular diseases		p-value
		Present	Absent	
How many hours of sleep do you have on average in 24 hours?	0-4 hours	18 (24.6%)	55 (75.4%)	0.041
	5-6 hours	7 (10.1%)	62 (89.9%)	
	7-8 hours	1 (7.1%)	13 (92.9%)	
Are you suffering from sleep problems?	Yes	5 (17.9%)	27 (82.1%)	0.048
	No	19 (16.2%)	98 (83.8%)	
How severe do you consider your sleep problems to be?	Absent	5 (5.2%)	92 (94.8%)	0.00001
	Mild	14 (36.8%)	24 (63.15%)	
	Moderate	5 (29.5%)	12 (70.5%)	
	Severe	2 (50%)	2 (50%)	

## Discussion

A healthy amount of sleep each night is necessary for preserving a healthy heart. Irregular sleep patterns or insufficient sleep can pose a threat to heart health. It is widely known that lack of sleep can negatively impact overall health as well as the biological systems that manage it, including glucose metabolism, blood pressure, and inflammation [11]. The consequences of chronic sleep deprivation are significant and can have long-term implications for our health, increasing the risk of developing chronic illnesses [13]. The study aimed to investigate the factors, allied situations, and behaviors that influence sleep among young adults and determine an association between sleep and the risk of cardiovascular disease. A total of 156 individuals participated in the study, with 58.3% being male and 41.7% female. The age range of the subjects was 18 to 24 years, with a mean age of 21.65 ± 1.76 years for participants with CVD. A study conducted by Gupta et al. on age-specific trends in cardiovascular risk factors among adolescents and young adults, focusing on a similar age group, demonstrated that cardiovascular risk factors increase exponentially with age, particularly after individuals in India reach the age group of 30 to 39 years [6].

An analogous study conducted by Singh et al. in New Delhi, India, on a total of 510 students, of whom 57.47% were males and 42.53% were females, ranging in age from 12 to 18 years, found that 7.84% of them had systolic hypertension and showed the influence of age on blood pressure [14]. Another study by Chakraborty et al. conducted in four urban schools in Kolkata, India, with a total of 979 students, reported a prevalence of childhood hypertension of 1.53% and showed a significant association between high blood pressure and increasing age [15].

The findings of the current study reveal a significant association between sleep duration, the prevalence of sleep problems, and CVDs. Our results (Table 3) provide important insights into the association between key factors, behaviors, and situations influencing sleep and CVD among the study participants (n=156). The analysis reveals statistically significant associations between sleep duration, sleep problems, and the presence of CVDs. First, considering the duration of sleep in study participants, those who reported having fewer hours of sleep (0-4 hours) exhibited a higher prevalence of CVDs compared to those with longer sleep durations. Specifically, 24.6% of individuals with shorter sleep durations had CVDs, while 92.9% of those with longer sleep durations were free from such conditions.

**Figure 1:**
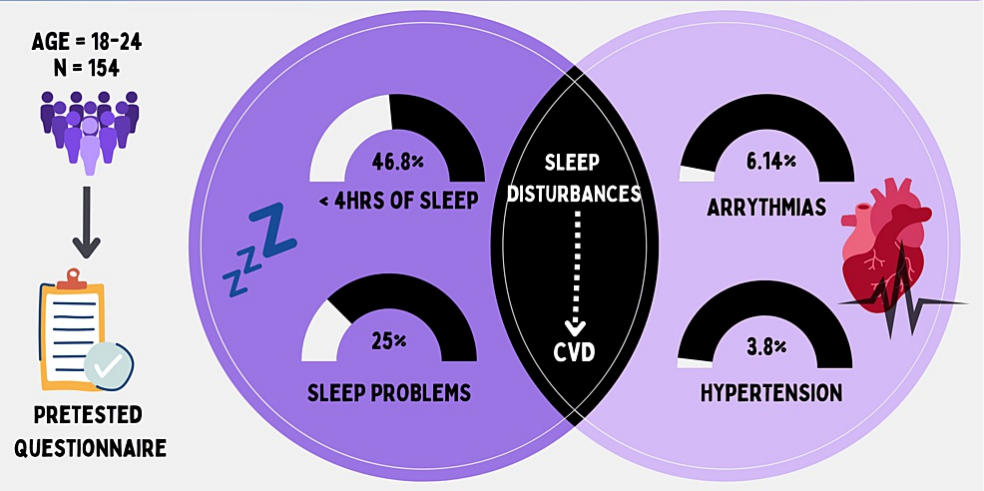
Association of sleep and cardiovascular morbidity

The presence of sleep problems also demonstrated a significant association with CVDs. Participants who reported having sleep problems had a higher prevalence of CVDs (17.9%) compared to those without sleep problems (16.2%). Furthermore, when assessing the severity of sleep problems, a clear association with the presence of CVDs was observed. A higher prevalence of CVDs was seen in individuals with moderate (29.5%) to severe (50%) sleep problems compared to those with no (5.2%) or mild (36.8%) sleep problems. This association has been highlighted in Figure 1.

Several analogous studies have shown that, in comparison to seven to eight hours of sleep, both shorter and longer sleep durations are related to risk factors for CVD. These risk factors include diabetes, high blood pressure, and obesity [16,17]. A similar study by Shankar et al. reported a correlation between insufficient rest or sleep and CVD (OR 1.55-1.79) [18]. Furthermore, it has been estimated that 7.5% of Asian Indians suffer from obstructive sleep apnea (OSA) in western India. There is mounting evidence that OSA is independently linked to obesity, hypertension, and an increased risk of CVD and mortality [19-21].

A study conducted by Chen et al. among 125 individuals with heart failure investigated self-reported health-related quality of life and sleep problems. The Pittsburgh sleep quality index (PSQI) was used, and the mean score was 9.06 ± 0.93. The study categorized 74.4% of the participants as poor sleepers [22]. These findings corroborate the findings of our study and strongly support the idea that insufficient sleep over an extended period is associated with an increased risk of acquiring CVDs.

**Figure 2:**
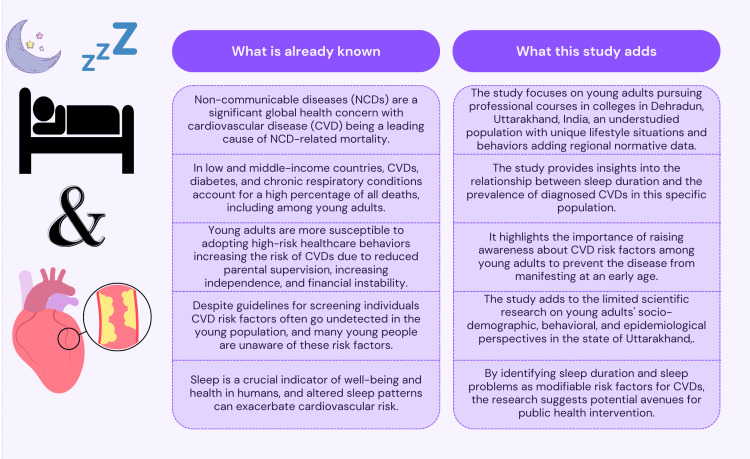
Summary of the study's findings

The research question addressed in the present study is novel, as there have been few studies conducted in a similar context with young adult students pursuing professional courses in Uttarakhand, India. To that end, the initiative, irrespective of the magnitude of the disease burden, is significant and will serve as an important precedent for further research considering the potential rise of lifestyle diseases across age groups. However, a few limitations of this study include a relatively small sample size, a cross-sectional study design, and the use of self-reported scales for data collection. Nevertheless, we believe that the study lays a good foundation for further large-sample cohort studies to further investigate the potential determinants of cardiovascular health in this age group. The contribution of this study is summarized in Figure 2.

## Conclusions

According to the findings of the study, we found that sleep duration and sleep problems were significantly associated with the prevalence of CVD in young adults. These findings suggest sleep duration (hours of sleep on an average of 24 hours) and sleep problems could be risk factors for CVDs. The majority of the factors influencing sleep that were detected among the participants of the study were modifiable; hence, they can be averted by modifying behavior and lifestyle. Effective health promotion activities have the potential to reverse the behavioral component influencing the development of CVD risk factors from a young age when social learning behaviors start developing. This has further implications for the prevention of impending cardiovascular events. The scope of the outcome of this sample study may not be limited to professional institution settings only. It is felt that a greater emphasis should be placed on community-endorsed behavior change communication (BCC) material and methods; community-based research studies for the development, implementation, and validation of public health interventions; and insight into innovative preventative strategies for control of CVDs among young adults.
